# Data on personal and contextual factors of university students on their entrepreneurial intentions in some Turkish universities

**DOI:** 10.1016/j.dib.2019.105086

**Published:** 2020-01-03

**Authors:** Harun Sesen, Mehmet Ali Ekemen

**Affiliations:** European University of Lefke, Turkey

**Keywords:** Entrepreneurial intention, Entrepreneurial self-efficacy, Locus of control, University, Knowledge about business, Turkey

## Abstract

This data examines the impact of personal (entrepreneurial self-efficacy and locus of control) and contextual (university environment and knowledge about business) factors on entrepreneurial intentions of university students in two Turkish universities. University students are one of the important groups to analyse entrepreneurial intentions. There are a number of researches that tested the effects of different personal and contextual factors separately on students' entrepreneurial intentions. This data tests the impacts of two different personal and contextual factors on students’ entrepreneurial intentions at the same time. Data collected using a questionnaire adapted from previous research. A quantitative research design is employed to test the data. The population of the data included 356 university students. Data was analysed running regression analyses with Statistical Package for Social Sciences (SPSS). Personal factors are confirmed stronger triggering factors than those of contextual.

**Table:** Specifications Table

Subject	Business, Management
Specific subject area	Business and Entrepreneurship
Type of data	Table
How data were acquired	Survey questionnaire
Data format	Raw Analysed
Parameters for data collection	University students at the beginning of their professional careers and working lives are seen as potential entrepreneurs. Working on how their individual features and the context they are taking education may affect their entrepreneurial intentions is an important research area. Thus, respondents of dataset were university students. The sampling method of this research was convenient sampling.
Description of data collection	Data was collected via printed surveys. Questionnaire is attached to the article.
Data source location	Institution: Gazi University and Baskent University City: Ankara Country: Turkey
Data accessibility	With the article

**Table:** 

**Value of the Data** • The data can be used to describe the effect of personal (entrepreneurial self-efficacy and locus of control) and contextual (university environment and knowledge about business) factors on the entrepreneurial intentions of university students in Turkey. • The dataset makes it possible to reorganize the entrepreneurship education given in the universities in a way to encourage the entrepreneurial intentions of university students. • This data present information on the importance of entrepreneurial self-efficacy on entrepreneurial intentions and weak effectiveness of university environment. • Policy makers and other university officials may consider this data as an information source that the university environment is not supportive enough to inspire entrepreneurial intentions of university students. • The data can be used to test the effects of different personal and contextual factors on students' entrepreneurial intentions.

## 1 Data description

The data was collected from university students of two Turkish higher education institutions. Total of five hundred (500) copies of surveys were distributed to two different universities, namely Gazi University and Baskent University located in city of Ankara, and totally three hundred and fifty-six (356) surveys were returned representing 71.2% response rate. Questionnaire and raw data are attached to the article as supplemental files. The data initiated descriptive cross-sectional design and surveys were distributed to the university students based on purposive, convenience sampling technique. Table 1 shows the total numbers of questionnaires which were distributed and collected in detail.

**Table 1:** Total questionnaire distributed and response rates.

Copies of questionnaires	Frequency		Response rate
	Number of copies of questionnaires distributed	Number of copies of questionnaires returned	
Gazi University	250	208	83.2%
Baskent University	250	148	59.2%
Total	500	356	71.2%

The data were designed to test the impacts of personal and contextual factors on the entrepreneurial intentions of university students. Personal factors in this data were entrepreneurial self-efficacy (ESE) and locus of control (LOC) while contextual factors were university environment (UE) and knowledge about the business (KAB). Entrepreneurial intention refers to the efforts of a student towards the behaviour of establishing his/her own business.

To test the hypotheses, two different regression analyses were employed. As shown on Table 2, Table 3, Table 4, first regression analysis was initiated to find out the impacts of personal factors on the entrepreneurial intentions of the students. Table 2 shows the model summary of the analysis based on first hypothesis which proposed personal factors’ effect on entrepreneurial intentions. The data showed that ESE and LOC explained 31.8% of the variance in entrepreneurial intentions (R square = 0.318, p < 0.05). Table 3 shows Analysis of Variance. The table shows the statistical significance of the result. The ANOVA table tests the null hypothesis to understand if it is significant. The model in this table is statistically significant (Sig = 000, p < 0.05) with the F- value of 82.445.

**Table 2:** Model summary. Source: Field survey 2016.

Model	R	R Square	Adjusted R Square	Std. Error of the Estimate	Change Statistics				
					R Square Change	F Change	df1	df2	Sig. F Change
1	,564^a^	,318	,315	1,32308	,318	82,445	2	353	,000

a Predictors: (Constant), ESE, LOC.

**Table 3:** ANOVA^b^. Source: Field survey 2016.

Model		Sum of Squares	df	Mean Square	F	Sig.
1	Regression	288,649	2	144,325	82,445	,000^a^
	Residual	617,945	353	1,751		
	Total	906,594	355			

a Predictors: (Constant), ESE, LOC.

b Dependent Variable: EI.

**Table 4:** Coefficients^a^. Source: Field survey 2016.

Model		Unstandardized Coefficients		Standardized Coefficients	t	Sig.
		B	Std. Error	Beta		
1	(Constant)	,157	,408		,384	,701
	LOC	,161	,073	,105	2,213	,028
	ESE	,757	,069	,518	10,958	,000

a Dependent Variable: EI.

Table 4 demonstrates which of the variables in the model contributed to the prediction of the dependent variable. The data show that both the LOC (Standardized Beta = 0.105, sig. = 0.028, p < 0.05) and ESE (Standardized Beta = 0.518, sig. = 0.000, p < 0.05) have statistically significant effects on the entrepreneurial intentions of the students, however, the contribution of ESE is stronger than that of LOC.

Table 5, Table 6, Table 7 show the second regression analyses which tested the effects of contextual factors on the entrepreneurial intentions of the students. Table 5 shows the model summary of the analysis based on second hypothesis which proposed contextual factors’ effect on entrepreneurial intentions. The data showed that KAB and UE explained 4.8% of the variance in entrepreneurial intentions (R square = 0.048, p < 0.05). Table 6 shows Analysis of Variance. The table demonstrates the statistical significance of the result. The model in this table is statistically significant (Sig = 000, p < 0.05) with the F- value of 8.872.

**Table 5:** Model summary. Source: Field survey 2016.

Model	R	R Square	Adjusted R Square	Std. Error of the Estimate	Change Statistics				
					R Square Change	F Change	df1	df2	Sig. F Change
1	,219^a^	,048	,042	1,56376	,048	8,872	2	353	,000

a Predictors: (Constant), UE, KAB.

**Table 6:** ANOVA^b^. Source: Field survey 2016.

Model		Sum of Squares	df	Mean Square	F	Sig.
1	Regression	43,390	2	21,695	8,872	,000^a^
	Residual	863,204	353	2,445		
	Total	906,594	355			

a Predictors: (Constant), UE, KAB.

b Dependent Variable: EI.

**Table 7:** Coefficients^a^. Source: Field survey 2016.

Model		Unstandardized Coefficients		Standardized Coefficients	t	Sig.
		B	Std. Error	Beta		
1	(Constant)	2,676	,555		4,819	,000
	KAB	,249	,088	,148	2,822	,005
	UE	,148	,053	,145	2,770	,006

a Dependent Variable: EI.

Table 7 shows which of the variables in the model contributed to the prediction of the dependent variable. The data indicate that both the KAB (Standardized Beta = 0.148, sig. = 0.005, p < 0.05) and UE (Standardized Beta = 0.145, sig. = 0.006, p < 0.05) have statistically significant effects on the entrepreneurial intentions of the students.

## 2 Experimental design, materials, and methods

The data was based on quantitative analysis. The method of analysing the data was regression analyses. The data was gathered from students in two selected Turkish universities. Five hundred questionnaires were distributed and three hundred and fifty-six were gathered. The data collecting instrument has been adapted from already used previous papers [[1], [2], [3], [4]]. Questions had 5-point Likert scale from strongly disagree (1) to strongly agree (5) which measures the respondents’ attitude to what extent they agree or disagree with the statement. Data were gathered from students who were volunteer during the course hours with the help of lecturers and students were given a brief information about the purpose of the study and the confidentiality. Data is processed using SPSS-25.
