# Supplementary material for: Data on personal and contextual factors of university students on their entrepreneurial intentions in some Turkish universities
Source: Data Brief. 2020 Jan 3;28:105086. doi: 10.1016/j.dib.2019.105086 (PMC6950781; doi:10.1016/j.dib.2019.105086)
Supplement: Multimedia component 1 [file mmc1.doc]

**Questionnaire of the study**

**Entrepreneurial intentions**

1. I am ready to do anything as an entrepreneur
2. My professional goal is to be an entrepreneur
3. I will make every effort to establish and maintain my own business
4. I am committed to starting a business in the future
5. I'm seriously thinking of starting my own business.
6. I have serious intentions to start my own business one day

**Entrepreneurial self-efficacy**

1. I think I have the leadership skills needed to start my own business
2. I think I have reached the level of mental maturity that I can establish my own business
3. I can generate new business ideas
4. I can develop new methods for production, marketing and management
5. I can capture opportunities for new products and services in the market
6. I can find new business areas with growth potential
7. I can act quickly to take advantage of opportunities
8. I can react quickly to unexpected situations
9. I do not disturb my morale when faced with negative situations

**Locus of control**

1. I believe perseverance and hard work often leads to success
2. I don't believe in luck at work
3. I have more control over events than luck
4. I never give up if I fail a job

**University environment**

1. My education at the university allows me to see the opportunities in the market
2. My university encourages students to progress towards their own ideas and ideals
3. My university education encourages me to start my own business
4. The creative atmosphere at my university encourages me to start a new business

**Business knowledge**

1. Those who start their own business must have a clear knowledge of technology and business environment
2. People who will start their own business must have sufficient experience and skills
3. I can access information that can help me starting my own business
